# The Impact of and Adaptations Due to the COVID-19 Pandemic on the Histopathological Diagnosis of Skin Pathologies, Including Non-Melanocyte and Melanoma Skin Cancers—A Single-Center Study in Romania

**DOI:** 10.3390/medicina57060533

**Published:** 2021-05-27

**Authors:** Iuliu-Gabriel Cocuz, Maria-Elena Cocuz, Raluca Niculescu, Mihaela Cornelia Șincu, Andreea Cătălina Tinca, Adrian-Horațiu Sabău, Diana Maria Chiorean, Silviu Horia Morariu, Ovidiu Simion Cotoi

**Affiliations:** 1 Doctoral School of Medicine and Pharmacy, George Emil Palade University of Medicine, Pharmacy, Sciences and Technology of Targu Mures, 540142 Targu Mures, Romania; iuliu.cocuz@umfst.ro (I.-G.C.); niculescuralu@yahoo.com (R.N.); mihaela.sincu02@gmail.com (M.C.Ș.); andreeatinca93@gmail.com (A.C.T.); sabauhoratiu@gmail.com (A.-H.S.); 2 Pathology Department, Mures Clinical County Hospital, 540011 Targu Mures, Romania; chioreandianamaria@yahoo.com (D.M.C.); ovidiu.cotoi@umfst.ro (O.S.C.); 3 Fundamental Prophylactic and Clinical Disciplines Department, Faculty of Medicine, Transilvania University of Brasov, 500003 Brașov, Romania; 4 Clinical Infectious Diseases Hospital of Brasov, 500174 Brasov, Romania; 5 Dermatology Department, George Emil Palade University of Medicine, Pharmacy, Sciences and Technology of Targu Mures, 540142 Targu Mures, Romania; silviu.morariu@umfst.ro; 6 Pathophysiology Department, George Emil Palade University of Medicine, Pharmacy, Sciences and Technology of Targu Mures, 540142 Targu Mures, Romania

**Keywords:** COVID-19 pandemic, non-melanocytic cancers, histopathology

## Abstract

*Background and Objectives*: The COVID-19 pandemic has globally affected health systems and services. Non-melanoma skin cancers (NMSCs) are the most common malignancies around the world. This study aimed to analyze the differences in the benign and malignant histopathological diagnoses performed on radical excision skin tumors and skin biopsies in the dermatopathology ward in Mures Clinical County Hospital, Targu Mures, Romania, 1 year prior to and during the COVID-19 pandemic, to emphasize the changes in the diagnostic process as per the new regulations. *Materials and Methods:* A total of 1168 histopathological diagnoses were included in the study—302 from the COVID-19 period and 866 from the non-COVID-19 period—considering the number, type, and frequency of the histopathological diagnoses as variables to be analyzed. *Results*: In the COVID-19 period, out of the 55 NMSC and melanoma histopathological diagnoses, 50.9% (*n* = 28) were BCCs, 20% (*n* = 11) were SCCs, 10.9% (*n* = 6) were basosquamous cell carcinomas, and 18.18% (*n* = 10) were melanoma cases. Regarding the non-COVID-19 period, out of the 173 NMSC and melanoma histopathological diagnoses, 46.82% (*n* = 81) were BCCs, 22.54% (*n* = 39) were SCCs, 7.51% (*n* = 13) were basosquamous cell carcinomas, and 23.12% (*n* = 40) were melanoma cases. *Conclusions:* During the COVID-19 pandemic, a decrease in histopathological diagnoses at the dermatopathology ward in our hospital was observed, for both benign and malignant pathologies, especially for NMSCs and melanomas, compared to the same period 1 year prior to the pandemic.

## 1. Introduction

Many patients globally suffered respiratory symptoms at the end of 2019 and needed intensive care. The identified virus, SARS-CoV-2, which is the cause of the COVID-19 disease, spread rapidly throughout the world [1]. In less than 2 months, most countries were affected by the disease. The World Health Organization (WHO) declared the outbreak of COVID-19 a pandemic, which has caused major health challenges worldwide [2]. There were over 133 million confirmed cases and over 2.9 million COVID-19 deaths worldwide as of 5 April 2021 [3].

Cutaneous lesions can be divided into three main categories: benign lesions, malignant lesions, and premalignant lesions. In histopathology, lesions can be identified on the basis of the primary cell of origin of the lesion or which of the three components of the skin is most affected by the pathologic process (epidermis, dermis, and hypodermis). The majority of skin lesions are benign and, morphologically, they appear as various dermatitis, nodules, cystic lesions, keratotic lesions, or papules that grow slowly. Malignant tumors of the skin are solitary lesions with irregular and rapidly growing dimensions that may ulcerate. These tumors of the skin are able to metastasize and are able to appear as new lesions or from other pre-existing skin lesions, being divided into melanocytic and non-melanocytic skin cancers. Histopathological diagnostics and treatments must be in accordance with the clinical presentation and type of surgery: biopsy or elective surgery [4,5].

Non-melanocyte skin cancers (NMSCs), also known as keratinocyte cancers, are the most common and most frequently diagnosed types of cancer in humans. Various NMSCs have been reported in the literature, with various histologic versions that frequently cause important differential diagnoses with other cutaneous tumors [6,7]. Compared to other malignancies, non-melanocytic skin cancers express low metastatic potential and are typically associated with a favorable prognosis. While their metastatic potential is limited, these malignancies can be exceedingly destructive to local tissue, leading to some patients requiring complex excisional and reconstructive procedures [8]. Despite the appearance of novel nonsurgical treatment modalities, surgical resection remains the most common treatment method for NMSCs, with 4 mm clinical margins in low-risk basal cell carcinomas (BCCs) and 4–6 mm clinical margins in local low-risk squamous carcinomas (SCCs). Melanocytic skin cancer, also known as melanoma, is one of the most aggressive types of skin cancer and one of the leading causes of cancer-related mortality due to its metastatic power. The majority of patients with newly diagnosed melanoma are in the early stage of the disease. For these patients, surgical excision is the treatment of choice and is curative in the majority of cases. When diagnosed at an advanced stage, melanoma remains a lethal type of cancer [9].

The COVID-19 pandemic has highly diminished opportunities to perform elective surgery for skin cancers in plastic surgery wards. In order to establish a priority in performing elective surgery in surgical wards, some measures should be taken into consideration, with one of the most used measures today being the risk of worsening of the skin lesion within the next month. Additionally, patients who had their melanoma excised recently or patients with ulcerated and bleeding skin cancers should be taken into consideration and prioritized in order to benefit from urgent and adequate treatment during the COVID-19 period. Furthermore, elective surgery should be considered for rapidly growing lesions such as basal cell carcinomas or squamous cell carcinomas [10].

This study aimed to analyze the differences in the histopathological diagnoses established by H&E staining and immunohistochemistry performed on radically excised skin tumors and skin biopsies in the dermatopathology ward in Mures Clinical County Hospital, Romania, both benign and malignant, 1 year prior to and during the COVID-19 pandemic, to emphasize the changes in the diagnostic process and number of histopathological diagnoses as per the new regulations.

## 2. Materials and Methods

A cross-sectional study was performed by analyzing the diagnostic data from the dermatopathology ward in Mures Clinical County Hospital, Targu Mures, Romania, between April 2019 and February 2020 (before the COVID-19 pandemic) and between April 2020 and February 2021 (during the COVID-19 pandemic). The hospital in which the study was performed is a county university hospital, which has both clinical and surgical wards. The samples came to our dermatopathology ward from the surgical units, especially the plastic and reconstructive surgical ward and the general surgery wards for patients who underwent radical excision, as well as from the dermatology clinic for patients who underwent skin punch biopsies. The two study periods were defined as the COVID-19 pandemic period (April 2020–February 2021) and the non-COVID-19 period (April 2019–February 2020). Diagnostic data were collected from the ward’s database and included histopathological reports, especially histopathological diagnoses. After collecting the data, the number of histopathological diagnoses, the types of histopathological diagnoses, and the frequency of each histopathological diagnosis were analyzed and represented graphically using the Microsoft Office suite, specifically Microsoft Excel and Descriptive Statistics. The inclusion criteria comprised dermatopathology tissue samples that were sent from different surgical wards in our hospital that were analyzed and diagnosed during the study period. The exclusion criteria comprised cases with other histopathological diagnoses that were sent for consultation to the dermatopathology ward. According to these criteria, we included 1168 histopathological diagnoses and excluded nine histopathological diagnoses.

## 3. Results

**Figure 1:**
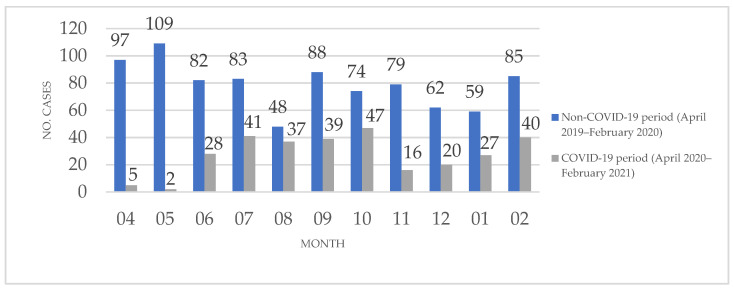
The monthly distribution of histopathological diagnoses established between April 2019 and February 2020 and between April 2020 and February 2021 in the dermatopathology ward of Mures Clinical County Hospital, Targu Mures, Romania.

Of the 1168 histopathological diagnoses included in the study, 302 were established during the COVID-19 period and 866 were established during the non-COVID-19 period. Figure 1 presents the monthly distribution of histopathological diagnoses by case numbers, which the dermatopathology ward established during the two periods.

The main histopathological diagnoses established during the COVID-19 period, based on the presence of benign, benign-appearing, and malignant lesions, as well as the number of cases for each pathology, are presented in Table 1.

**Figure 2:**
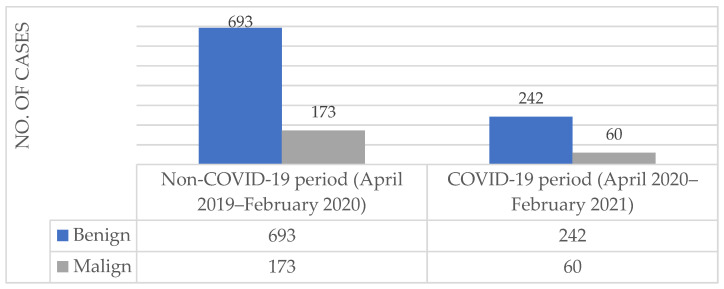
The counts of benign and malignant histopathological diagnoses established between April 2019 and February 2020 and between April 2020 and February 2021 in the dermatopathology ward of Mures Clinical County Hospital, Targu Mures, Romania.

A comparison between the benign and malignant lesions diagnosed during the COVID-19 and non-COVID-19 periods is presented in Figure 2. During the COVID-19 period, out of the 302 histopathological diagnoses, 80.13% (*n* = 242) of the cases presented with benign lesions and 19.87% (*n* = 60) presented with malignant lesions. In the non-COVID-19 period, out of the 866 histopathological diagnoses, 74.25% (*n* = 693) of the cases presented with benign lesions and 25.75% (*n* = 173) presented with malignant lesions.

**Figure 3:**
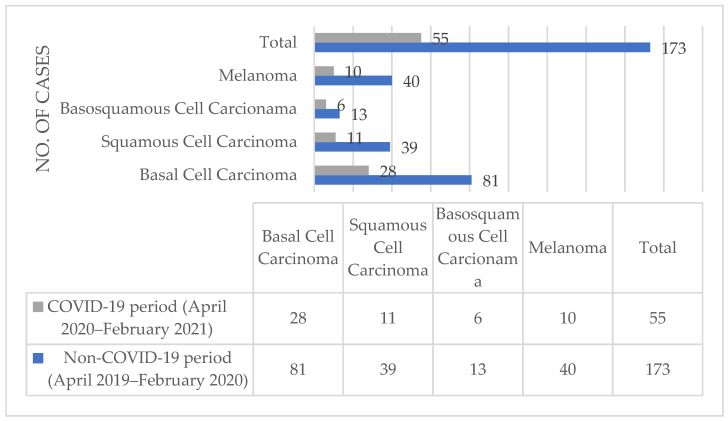
NMSC and melanoma counts from the histopathological diagnoses established between April 2019 and February 2020 and between April 2020 and February 2021 in the dermatopathology ward of Mures Clinical County Hospital, Targu Mures, Romania.

Regarding the malignant histopathological diagnoses, Figure 3 presents the distribution of the NMSCs, comprising BCCs, SCCs, and basosquamous carcinomas, as well as melanocytic skin cancers, comprising melanomas, during the two studied periods. In the COVID-19 period, out of the 55 NMSC and melanoma histopathological diagnoses, 50.9% (*n* = 28) of the cases presented with BCCs, 20% (*n* = 11) presented with SCCs, 10.9% (*n* = 6) presented with basosquamous cell carcinomas, and 18.18% (*n* = 10) presented with melanoma. Regarding the non-COVID-19 period, out of the 173 NMSC and melanoma histopathological diagnoses, 46.82% (*n* = 81) of the cases presented with BCCs, 22.54% (*n* = 39) presented with SCCs, 7.51% (*n* = 13) presented with basosquamous cell carcinomas, and 23.12% (*n* = 40) presented with melanoma.

## 4. Discussion

The COVID-19 pandemic caused by the outbreak of SARS-CoV-2, i.e., the novel coronavirus, emerged suddenly and has become a global health problem, as declared by the WHO in March 2020. Every country has established new procedures and protocols for the treatment and diagnosis of various pathologies, dividing their national health facilities into COVID-19 and non-COVID-19 wards. Due to the sudden appearance of this epidemiological emergency in the form of a pandemic, the restrictions imposed due the characteristics of the epidemiological event (in this case, COVID-19, a disease with airborne transmission and a high risk of contagion), and the necessity to move almost the whole healthcare force toward providing COVID-19-related services, the clinical and surgical activities of many specialists have declined, influencing patients’ access to high-quality medical services and decreasing patients’ satisfaction with healthcare referrals, thus influencing the quality of life for patients diagnosed with skin cancers [11].

Pathology services include training and teaching for residents, autopsies, and quality assurance in the activity of the laboratory, combined with diagnostic activities. The COVID-19 pandemic has challenged all of these aspects. As a result of the reduction in surgical procedures and an almost complete cessation of aerosol-generating specimens and procedures, such as gastric or pulmonary endoscopy, the diagnostic workload and workflow have been dramatically affected in almost all histopathology services since the beginning of the COVID-19 pandemic [1,12].

Throughout the COVID-19 pandemic, a series of functioning rules have been implemented, not only internationally but also in our country. These proceedings had a much greater applicability at the level of clinical departments; however, some of the guidelines also applied to paraclinical departments. Diagnostic procedures in a pathology wards can be categorized as pre-analytical, analytical, and post-analytical. Regarding the possible transmission of SARS-CoV-2, the pre-analytical phase is the most favorable phase in which the virus can intervene. During this phase, fresh tissue is received in the pathology ward, and the tissue samples, biological materials, or formalin-fixed tissue and organs are processed. According to the guidelines from RCPath (Royal College of Pathologists), fresh tissue handling is discouraged [1,13]. The measures taken to minimize the possible SARS-CoV-2 transmission route while handling the tissue samples are aimed at increasing the time of the formalin fixation process. Additionally, while handling the tissue samples, the laboratory personnel are advised to wear adequate protective equipment. Another applied measure involved establishing a program for receiving the biological samples arriving from the surgical departments. As in those cases reported in the literature, in our pathology service, there were no cases of infection with SARS-CoV-2 related to tissue sample handling [1,2]. The personnel from our ward are tested for COVID-19 frequently, and almost 95% were vaccinated with a COVID-19 approved vaccination scheme before the end of March 2021. The dermatopathology ward in Mures Clinical County Hospital has been strongly affected by these changes. The number of histopathological diagnoses significantly decreased from 866 histopathological diagnoses during the non-COVID-19 period to 302 during the COVID-19 period, as shown in Figure 1. Specifically, there was a large decrease in the first 2 months of the COVID-19 period (April and May 2020), with an increase in the subsequent months. Peaks were observed in August 2020 and October 2020, but they were not comparable to the COVID-19 period.

As seen in Table 1, the main histopathological diagnoses established in our ward were based on benign pathologies, most of which were epidermoid cysts, granulation tissues, and junctional and dermal nevi. Additionally, biopsies from the skin were used to establish diagnoses of psoriasis or psoriasiform dermatitis in a significant number of cases. Regarding the comparison between the benign and malignant diagnoses established in our ward, a significant decrease was observed in both categories. The malignant diagnoses during the COVID-19 period decreased to less than half of the diagnoses from the non-COVID-19 period. Even though the number of malignant diagnoses decreased, as seen in Figure 3, there was a slight difference in the percentages of BCC and SCC diagnoses out of the total malignant diagnoses. A greater difference was seen in the diagnoses of BCC between the two study periods, with an increase during the COVID-19 period. Melanoma diagnoses decreased to one-quarter during the COVID-19 pandemic compared to the diagnoses established during the non-COVID-19 period. Moreover, the histopathological diagnoses of NMSCs were negatively impacted during the COVID-19 period, with fewer cases diagnosed during this period.

During the COVID-19 pandemic, several multidisciplinary recommendations were developed regarding the local treatment of skin cancer patients. Regarding NMSCs, specifically, BCCs and SCCs, the main recommendations were as follows: to delay the treatment for a period of 2 or 3 months, unless the patient is highly symptomatic or immunosuppressed, or to prioritize those with an advanced stage of disease (≥T2b). Concerning those patients suffering from Merkel cell carcinoma, the main recommendations suggest the prioritization of treatment or the possibility of deferral for those with a favorable stage of disease (T1b) who are at a high risk of experiencing COVID-19 complications. Regarding cutaneous melanoma, the recommendations suggest delaying treatment for patients with T0–T1 stages of the disease for 3 months if no macroscopic residual disease is found when a biopsy is performed. For those patients with disease beyond stage T2, a 3 month delay is recommended if the biopsy margins are declared negative [14,15].

Considering the specific area in which pathology services work, a series of protective measures have been taken. Due to the new social distancing regulations, the working schedule has been adapted in order to respect the national regulations. Residents in training have changed their schedule according to their supervisor. If there is a case of direct contact with a SARS-CoV-2-positive person, social distancing is immediately implemented and the national regulations for testing and quarantine are applied. The “working from home” concept has been adopted by many domains during the pandemic. In the pathology ward, face-to-face meetings are only available for the pre-analytical phase while handling fresh tissue, as well as the analytical phase, in which slides are analyzed using a double-header or multi-header microscope that can be set in order to establish a pathologist–resident connection and that allows for focusing on hands-on training. The post-analytical phase is performed mostly from home, via online training and meetings [1,16].

In our pathology service, there was less experience with working from home before the COVID-19 pandemic. For this reason, we implemented a secure connection in order to be able to assure that the residents’ training and personal training were in accordance with the new regulations by using dedicated e-learning platforms and online communication software [1,17].

The COVID-19 pandemic has had a negative effect on those patients who needed surgery, especially the oncological patients. Overcoming the fear of infection and, most importantly, returning to a normal life will allow patients to receive medical consultation and go to the dermatologist. We expect to observe an increase in the number of dermatological cases and, particularly, an increase in the number of NMSC and melanoma cases in the near future. We are also expecting to notice cases in much more advanced stages due to the period in which patients did not receive medical consultation regarding their skin pathology.

## 5. Conclusions

During the COVID-19 pandemic, a decrease in histopathological diagnoses at the dermatopathology ward in our hospital was observed, for both benign and malignant pathologies, especially NMSCs and melanomas, compared to the same period 1 year prior to the pandemic. The COVID-19 pandemic has been a challenge for histopathology services. In terms of maintaining the high quality of histopathological diagnostics, keeping a social distance, and adapting the patterns and processes of work, a restructuring of the activity in every part of the laboratory was needed. By using the new regulations, we were able to limit the transmission of SARS-CoV-2 without changing the quality of the histopathological diagnoses established in our ward, thus maintaining the patients’ satisfaction regarding the medical services that they need for diagnostics.

## Figures and Tables

**Table 1:** The histopathological diagnoses established between April 2020 and February 2021 in the dermatopathology ward of Mures Clinical County Hospital, Targu Mures, Romania.

Benign Lesions			
Histopathological diagnosis	No. of Cases	Histopathological Diagnosis	No. of Cases
Chalazion	3	Lobular capillary hemangioma	1
Clavus	6	Mixed mesenchymal tumor	2
Dermal naevus	19	Mixed thrombus	1
Dermatofibroma	6	Normal skin	5
Dilated pore of winer	2	Pseudoepitheliomatous hyperplasia	1
Epidermoid cyst	33	Psoriasiform dermatitis	4
Fibrolipoma	2	Psoriasis	12
Fibroma	5	Pyoderma vegetans	1
Gigantocellular granuloma	4	Pyogenic granuloma	3
Granulation tissue	29	Squamous papilloma	16
Hyperkeratosis	1	Seborrheic keratosis	9
Incarnated nail	3	Sinusoidal hemangioma	7
Junctional naevus	21	Skin atrophy	2
Keloid scar	3	Spitz naevus	1
Keratoacanthoma	7	Sub-chronic dermatitis	1
Lentigo	1	Trichilemmal cyst	12
Lichen sclerosus	1	Trichoepithelioma	1
Lymphatic nodules	4	Verrucous naevus	4
Lipoma	10	Vesiculobullous lesion	1
**Total Cases: 242**			
**Malignant Lesions**			
Histopathological diagnosis	No. of Cases	Histopathological Diagnosis	No. of Cases
BCC	28	Porocarcinoma	1
Basosquamous carcinoma	6	Sarcoma	4
Melanoma	10	Squamous carcinoma (SCC)	11
**Total Cases: 60**			

## Data Availability

All data produced here are available and can be produced upon request.
